# Comparative incidence and burden of respiratory viruses associated with hospitalization in adults in New York City

**DOI:** 10.1111/irv.12842

**Published:** 2021-01-26

**Authors:** William D. Sieling, Connor R. Goldman, Matthew Oberhardt, Matthew Phillips, Lyn Finelli, Lisa Saiman

**Affiliations:** ^1^ Department of Pediatrics Columbia University Irving Medical Center New York NY USA; ^2^ Value Institute New York‐Presbyterian Hospital New York NY USA; ^3^ Center for Observational and Real‐World Evidence Merck & Co., Inc. Kenilworth NJ USA; ^4^ Department of Infection Prevention & Control NewYork‐Presbyterian Hospital New York NY USA; ^5^ Present address: University of Minnesota Medical School Duluth MN USA; ^6^ Present address: Center for Observational and Real‐World Evidence Merck & Co., Inc. Kenilworth NJ USA

**Keywords:** adenovirus, human coronaviruses, influenza, parainfluenza viruses, respiratory syncytial virus

## Abstract

**Background:**

Although the burden of influenza is well characterized, the burden of community‐onset non‐influenza respiratory viruses has not been systematically assessed. Understanding the severity and seasonality of non‐influenza viruses, including human coronaviruses, will provide a better understanding of the overall disease burden from respiratory viruses that could better inform resource utilization for hospitals and highlight the value of preventative strategies, including vaccines.

**Methods:**

From October 2017 to September 2019, a retrospective study was performed in a pre‐defined catchment area to estimate the population‐based incidence of community‐onset respiratory viruses associated with hospitalization. Included patients were ≥18 years old, resided in New York City, were hospitalized for ≥24 hours, and had a respiratory virus detected within 3 calendar‐days of admission. Disease burden was measured by hospital length of stay (LOS), intensive care unit (ICU) admissions, and in‐hospital mortality and compared among those with laboratory‐confirmed influenza versus those with laboratory‐confirmed non‐influenza viruses (human coronaviruses, parainfluenza viruses, respiratory syncytial virus, human metapneumovirus, and adenovirus).

**Results:**

During the study period, 4232 eligible patients were identified of whom 50.9% were ≥65 years of age. For each virus, the population‐based incidence was highest for those ≥80 years of age. When compared to those with influenza viruses detected, those with non‐influenza respiratory viruses detected (combined) had higher population‐based incidence, significantly more ICU admissions, and higher in‐house mortality.

**Conclusions:**

The burden of non‐influenza respiratory viruses for hospitalized adults is substantial. Prevention and treatment strategies are needed for non‐influenza respiratory viruses, particularly for older adults.

## 1 BACKGROUND

Respiratory viruses cause significant morbidity and mortality in adults, especially among frail older adults and those with chronic comorbid conditions.^1^, ^2^ While the burden of influenza viruses is best studied, non‐influenza viruses such as respiratory syncytial virus (RSV), human metapneumovirus (hMPV), human coronaviruses (CoV), adenoviruses (AV), and parainfluenza viruses (PIV) are responsible for a substantial burden of illness in adults.^3^, ^4^, ^5^, ^6^

However, the population‐based incidence and burden of community‐onset non‐influenza respiratory viruses associated with hospitalization in adults have not been systematically assessed. Previous approaches have utilized weekly laboratory surveillance and syndromic surveillance associated with discharge data in statistical models to estimate rates of hospitalization and mortality associated with respiratory viruses.^4^, ^7^, ^8^ Yet, these methods may lack precision as specific viruses associated with acute respiratory infections (ARIs) are not routinely laboratory‐confirmed nor consistently reported in discharge records. Thus, the aims of this study were to estimate the population‐based incidence of different laboratory‐confirmed respiratory viruses in hospitalized adults and to describe the disease burden associated with different viruses measured by hospital length of stay (LOS), intensive care unit (ICU) admissions, and in‐hospital mortality. These parameters were compared in patients with influenza versus patients with non‐influenza respiratory viruses in efforts to provide an enhanced understanding of the overall disease burden from respiratory viruses and better inform resource utilization for hospitals and highlight the value of preventative strategies, including vaccines.

## 2 METHODS

### 2.1 Study design, sites, and subjects

From October 2017 to September 2019, a retrospective study was performed to identify hospitalized adults who had respiratory viruses detected from nasopharyngeal swab specimens using a multiplex polymerase chain reaction (mPCR) assay (described below). Study sites are located in Northern Manhattan, are part of the same academically affiliated hospital system, and include three hospitals: a ~750‐bed tertiary care hospital caring for adults, a ~200‐bed community hospital caring for adults, and a ~250‐bed children's hospital.

To estimate the population‐based incidence of community‐onset respiratory viruses associated with hospitalization, eligible patients were 18 years of age and older, resided in New York City, as defined by the zip code of their residence, were hospitalized for at least 24 hours, and had a respiratory virus detected within 3 calendar‐days of admission. Patients were identified from the electronic health record (EHR). The Columbia University Irving Medical Center institutional review board approved this study with a waiver of informed consent.

### 2.2 Viral detection

As part of the standard of care, patients with ARI symptoms who are being admitted are tested for respiratory viruses to inform appropriate transmission precautions and antiviral therapy for influenza. The study sites use the FilmArray Respiratory Panel (BioFire Diagnostics, Inc) which detects influenza (types A H3, A H1, B), PIV types 1‐4, RSV, hMPV, AV, rhinovirus/enterovirus (RV/EV), and human coronaviruses (CoV types 229E, HKU1, NL63, OC43).

### 2.3 Data analysis

Two one‐year periods, October 2017 to September 2018 (year 1) and October 2018 to September 2019 (year 2), were studied to assess seasonal differences in population‐based incidence and clinical burden for the respiratory viruses detected. To calculate population‐based incidence per 100 000 persons for each virus, the number of adults with a laboratory‐confirmed respiratory virus was divided by the adjusted 2010 US Census population estimate for the catchment area. Population‐based incidence and 95% confidence intervals were calculated overall and for four age strata: 18‐49, 50‐64, 65‐79, and ≥80 years of age.^9^ If more than one virus was detected in a single specimen, each virus contributed to the respective incidence calculation. The population estimate was adjusted by the hospitals’ percent market share for zip codes as determined by the New York Statewide Planning and Research Cooperative System (SPARCS).^10^ To improve the incidence estimate's reliability, the catchment area was defined as the eight zip codes in which the hospitals had ≥ 60% market share (10 032, 10 033, 10 034, 10 040, 10 452, 10 453, 10 463 and 10 471).

To assess the clinical burden of specific viruses, the following outcomes associated with each virus were determined: the median hospital LOS, the proportion of patients who had an ICU admission, the ICU LOS, and in‐hospital all‐cause mortality. Time spent in the emergency department prior to being admitted to an inpatient unit was included in LOS calculations. If more than one virus was detected in a single specimen, each virus contributed to the respective median LOS, ICU admission and ICU LOS, and in‐hospital mortality, which was determined from recorded death notes in the EHR. Two sample tests of proportions were used to compare outcomes (ICU admissions and mortality) between each virus and all others (eg, adenovirus vs. all other viruses). Median hospital LOS and median ICU LOS were compared using the Mann‐Whitney test for each virus vs. all others.

The population‐based incidence and clinical burden of influenza viruses (combined A H1, A H3, and B) were compared with those of non‐influenza viruses (combined CoV229E, CoVHKU1, CoVNL63, CoVOC43, PIV types 1‐4, RSV, hMPV, and AV) using the two‐sample test of proportions. RV/EV were excluded from the comparative analyses of the burden of influenza vs. non‐influenza viruses. Patients with co‐detections of influenza and non‐influenza viruses were excluded from these comparative analyses.

## 3 RESULTS

### 3.1 Study population

During the study period, 4232 adults were hospitalized with a laboratory‐confirmed respiratory virus and met the community‐onset case definition. Testing for a respiratory virus occurred on hospital day 1 for 90.7% of patients, on hospital day 2 for 5.0% of patients, and on hospital day 3 for 2.0% of patients, and 2.3% of patients had testing performed in ambulatory settings affiliated with the hospitals.

Selected demographic characteristics and the distribution of the zip codes of patients are shown in Table 1. Demographic characteristics were similar in year 1 and year 2; 50.9% of patients were ≥65 years of age, and 23.5% of patients were ≥80 years of age. Overall, 2447 (57.8%) of patients resided in zip codes that represented ≥ 60% of the hospital's market share.

**TABLE 1:** Selected characteristics of patients and detected respiratory viruses

	Year 1 October 2017‐September 2018 N = 2043	Year 2 October 2018‐September 2019 N = 2189	Total N = 4232
Demographic characteristics			
Age in years, n (%)			
18‐49	455 (22.3%)	526 (24.0%)	981 (23.2%)
50‐64	542 (26.5%)	555 (25.4%)	1097 (25.9%)
65‐79	534 (26.14%)	626 (28.6%)	1160 (27.4%)
>80	512 (25.1%)	482 (22.0%)	994 (23.5%)
Mean age (range)	63.5 (18‐110)	62.3 (18‐104)	62.8 (18‐110)
Female, n (%)	1144 (56.0%)	1247 (57.0%)	2391 (56.5%)
Hospital, n (%)			
A	1322 (64.7%)	1526 (69.7%)	2848 (67.3%)
B	644 (31.5%)	583 (26.6%)	1227 (29.0%)
C	77 (3.8%)	80 (3.7%)	157 (3.7%)
Reside in zip codes, n (%)			
>60% market share^a^	1181 (57.8%)	1266 (57.8%)	2447 (57.8%)
Reside in zip codes, n (%)			
<60% market share^b^	862 (42.2%)	923 (42.2%)	1785 (42.2%)
Respiratory viruses detected,^c^ n (%)			
Influenza (all)	503 (23.3%)	401 (17.3%)	904 (20.2%)
Influenza type AH3	270 (12.5%)	161 (6.9%)	431 (9.6%)
Influenza type AH1	48 (2.2%)	215 (9.3%)	263 (5.9%)
Influenza type B	173 (8.0%)	14 (0.6%)	187 (4.2%)
Influenza A equivocal	12 (0.6%)	11 (0.5%)	23 (0.5%)
Rhinovirus/enterovirus	765 (35.5%)	902 (38.8%)	1667 (37.2%)
Human coronavirus (all)	277 (12.8%)	366 (15.7%)	643 (14.4%)
Coronavirus type 229E	20 (0.9%)	126 (5.4%)	146 (3.3%)
Coronavirus type HKU1	119 (5.5%)	30 (1.3%)	149 (3.3%)
Coronavirus type NL63	68 (3.2%)	59 (2.5%)	127 (2.8%)
Coronavirus type OC43	70 (3.2)	151 (6.5%)	221 (4.9%)
Respiratory syncytial virus	198 (9.2%)	243 (10.5%)	441 (9.8%)
Parainfluenza viruses 1‐4	193 (8.9%)	175 (7.5%)	368 (8.2%)
Human metapneumovirus	182 (8.4%)	181 (7.8%)	363 (8.1%)
Adenovirus	39 (1.8%)	54 (2.3%)	93 (2.1%)

^a^ Represents eight unique zip codes used for population‐based incidence estimate.

^b^ Represents 130 and 133 unique zip codes in Year 1 and Year 2, respectively.

^c^ Includes specimens with more than one virus detected.

### 3.2 Viruses detection

**FIGURE 1:**
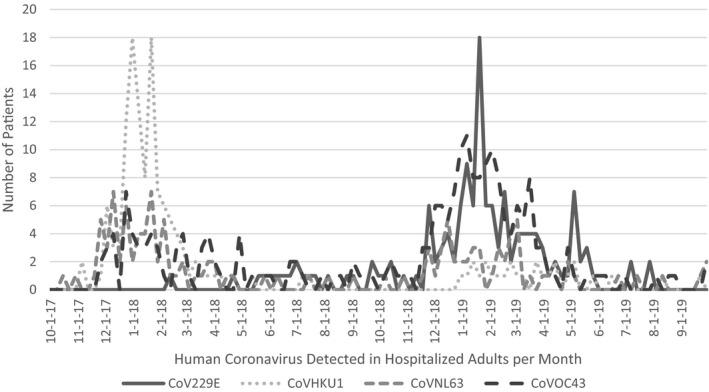
Epidemiologic Curve of Coronaviruses from October 2017 to October 2019. The number of patients hospitalized with the human coronaviruses (CoV) 229E, HKU1, NL63, and OC43 during the study period are shown. Human coronavirus HKU1 was most common during the winter months of year 1, and human coronavirus 229E was most common during the winter months of year 2. Human coronavirus 229E was not detected until March 2018

The proportion of patients who had specific viruses detected is shown in Table 1. Overall, 904 patients had influenza detected, 1667 had RV/EV detected, and 2551 had non‐influenza, non‐RV/EV respiratory viruses detected. The relative proportion of each virus was similar in year 1 vs. year 2 with the exception of the influenza viruses (A H1, A H3, and B) and the human coronavirus types. Influenza A H3 and influenza B were most common in year 1, while influenza A H1 and influenza A H3 were most common in year 2. The cumulative proportion of all coronaviruses was similar in both years, but seasonal differences occurred in the relative proportions of CoV229E, CoVHKU1, and CoVOC43 as illustrated in Figure 1. In year 1, CoVHKU1 was most common and had a sharp winter peak. In year 2, CoV229E peaked in winter, but continued to be detected throughout the spring.

Overall, co‐detection of two or more respiratory viruses occurred in 251 patients. In year 1, 116 (5.7%) of 2043 patients had co‐detection and RV/EV was detected in 46 (39.7%) of these 116 co‐detections. In year 2, 135 (6.2%) of 2189 patients had co‐detections and RV/EV was detected in 72 (53.3%) of these 135 co‐detections. Among the 251 total co‐detections, an Influenza virus and a non‐influenza virus were detected in 67 (26.7%).

### 3.3 Population‐based Incidence

**FIGURE 2:**
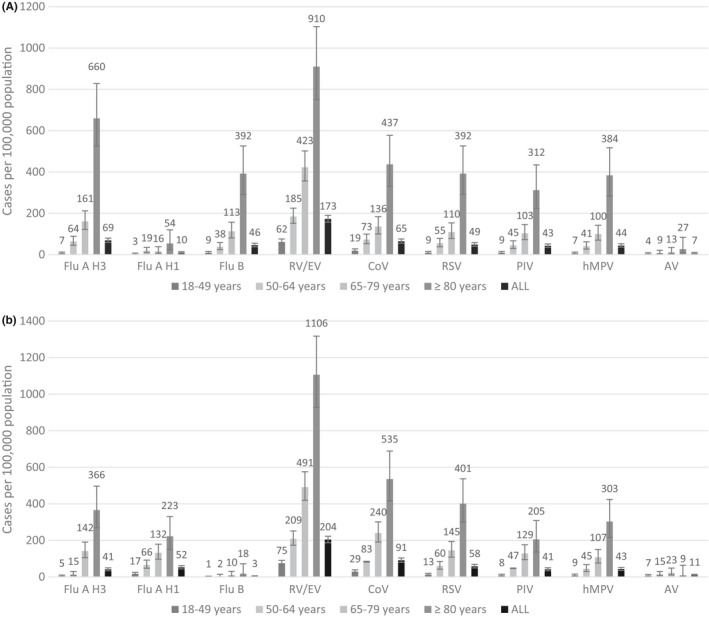
Year 1 (Figure a) and Year 2 (Figure b) Comparative Population‐based Incidence of Respiratory Viruses by Age Strata. The incidence of each virus per 100 000 persons (95% confidence intervals) overall and by the three age strata 18‐49, 50‐64, 65‐79, and >80 years of age are shown for year 1 and year 2. For both years and for every virus, the population‐based incidence was highest for those >80 years of age

The population‐based incidence for each virus overall and by age strata for year 1 and year 2 is shown in Figure 2A,B, respectively. Each year, RV/EV had the highest overall population‐based incidence estimates (in year 1, 173 hospitalizations and in year 2, 204 hospitalizations per 100 000 persons). After RV/EV, in year 1, influenza A H3 had the highest overall incidence (69 hospitalizations per 100 000 persons) followed by the combined CoV types (65 hospitalizations per 100 000 persons) and in year 2, the combined CoV types had the highest overall incidence (91 hospitalizations per 100 000 persons) followed by influenza A H1 (52 hospitalizations per 100 000 persons).

Incidence increased with age for all viruses for both years and those ≥80 years of age consistently had the highest incidence. The highest population‐based incidence in those ≥80 years of age for specific viruses was 660 hospitalizations per 100,000 persons for influenza A H3 in year 1 and 535 hospitalizations per 100 000 persons for the four combined human coronaviruses in year 2. Following RV/EV, the combined CoV types had the highest incidence estimates in both years among patients 18‐49 and 50‐64 years of age.

In year 1, the combined population‐based incidence of all influenza viruses (A H1, A H3, and B) for all age strata was significantly lower than the combined population‐based incidence of the non‐influenza respiratory viruses including CoV types, RSV, PIV types, hMPV, and AV (125 vs 208 hospitalizations per 100 000 persons, respectively, *P* < .001). Similarly, in year 2, the combined population‐based incidence of influenza viruses was significantly lower than that of these non‐influenza respiratory viruses (96 vs. 244 hospitalizations per 100 000 persons, respectively, *P* < .001).

### 3.4 Clinical burden associated with different viruses

The median LOS, the proportion of subjects admitted to an ICU, the ICU LOS, and in‐hospital mortality for each virus are shown in Table 2. As the burden was similar in each year, data for both years were combined. The overall median LOS was 4.2 days (interquartile range [IQR] 2.3, 7.8 days) and similar for each virus. Overall, 14.4% of patients had an ICU admission with a median ICU LOS of 4.0 days (IQR 2.1, 8.4 days). Compared with other viruses, CoV229E (*P* = .01) and AV (*P* = .02) were each associated with a significantly larger proportion of ICU admissions and AV was associated with the longest ICU LOS (*P* = .03). Of the 225 ICU admissions associated with RV/EV, 19 (8.4%) had another virus co‐detected. Four percent of patients died during hospitalization. RV/EV was associated with 31.8% of deaths, and CoV229E was associated with the highest mortality rate (12.3%, *P* < .001). The median LOS among those who died was 9.7 days (IQR 5.0, 19.8 days) and was longest for AV (38.0 days, *P* = .03).

**TABLE 2:** Morbidity and mortality associated with detection of different respiratory viruses

Virus	Patients n (%)	Median LOS, days (IQR)	ICU Admission n (%)	ICU Median LOS, days (IQR)	Crude Mortality n (%)	Median LOS of patients who died, days (IQR)
All influenza^a^	904 (20.2%)	3.9 (2.1, 7.1)	104 (11.5)	4.3 (2.2, 8.0)	29 (3.3)	8.7 (5.5, 15.7)
Flu A (H3)	431 (9.6%)	4.0 (2.2, 7.1)	47 (10.9)	3.8 (2.2, 6.8)	15 (3.5)	6.2 (5.1, 19.9)
Flu A (H1)	263 (5.9%)	3.4 (2.1, 6.9)	42 (16.0)	5.5 (2.2, 9.9)	9 (3.4)	8.5 (8.3, 19.8)
Flu B	187 (4.2%)	4.2 (2.1, 7.5)	15 (8.0)	3.5 (2.3, 6.2)	6 (3.2)	10.9 (10.0, 14.2)
RV/EV	1667 (37.2%)	4.1 (2.3, 7.6)	225 (13.5)	3.8 (2.1, 7.7)	54 (3.2)	6.6 (4.2, 18.4)
CoV229E	146 (3.3%)	4.4 (2.3, 8.1)	32 (21.9)	3.6 (1.7, 10.8)	18 (12.3)	11.0 (5.5, 24.2)
CoVHKU1	149 (3.3%)	4.3 (2.3, 8.7)	29 (19.5)	3.7 (1.9, 7.8)	14 (9.4)	7.8 (3.8, 12.1)
CoVNL63	127 (2.8%)	5.1 (3.0, 8.9)	26 (20.5)	3.3 (2.0, 7.6)	5 (3.9)	13.2 (11.3, 24.5)
CoVOC43	221 (4.9%)	4.0 (2.4, 7.8)	35 (15.8)	4.8 (2.0, 7.6)	8 (3.6)	8.1 (4.8, 11.6)
RSV	441 (9.8%)	4.4 (2.9, 8.3)	71 (16.1)	4.0 (2.1, 8.0)	23 (5.2)	8.1 (3.7, 25.5)
PIV	368 (8.2%)	4.2 (2.3, 8.1)	55 (14.9)	5.2 (2.6, 9.7)	18 (4.9)	10.8 (5.0, 18.9)
HMPV	363 (8.1%)	4.8 (2.7, 8.6)	60 (16.5)	4.4 (2.0, 8.5)	14 (3.9)	12.0 (8.6, 19.9)
AV	93 (2.1%)	5.1 (2.7, 11.2)	21 (22.6)	6.5 (3.6, 13.6)	7 (7.5)	38.0 (11.2, 39.0)
All^b^	4232 (100%)	4.2 (2.3, 7.8)	609 (14.4)	4.0 (2.1, 8.4)	171 (4.0)	9.7 (5.0, 19.8)

Abbreviations: AV, adenovirus; CoV, coronavirus; Flu, influenza; HMPV, human metapneumovirus; ICU, intensive care unit; IQR, interquartile range; LOS, length of stay; PIV, parainfluenza viruses; RSV, respiratory syncytial virus; RV/EV, rhinoviruses/enteroviruses.

^a^ Includes patients reported to have equivocal detection of influenza A.

^b^ Includes patients with more than one virus detected.

Those with RV/EV (n = 1,667 patients) and those with influenza/non‐influenza co‐detection (n = 67 patients) were excluded from the analysis comparing the burden of influenza viruses (784 patients) with that of the non‐influenza viruses (1714 patients). Compared to patients with influenza, a significantly higher proportion of patients with the non‐influenza viruses CoV, RSV, PIV, hMPV, and AV were admitted to the ICU (11.0% vs. 16.7%, respectively, *P* = .002). Compared to patients with influenza, a significantly higher proportion of patients with the non‐influenza respiratory viruses died (3.2% and 5.2%, respectively, *P* = .025).

## 4 DISCUSSION

This study provides further insights for the population‐based incidence of laboratory‐confirmed respiratory viruses associated with hospitalization in adults. Our findings may provide more precise estimates of the incidence of non‐influenza respiratory viruses than previous studies, which have often relied on diagnostic codes or syndromic surveillance, rather than systematic laboratory testing with a multiplex‐PCR assay, and thus may have underestimated the case burden.^11^ We also evaluated the incidence of respiratory viruses in the subset of patients ≥ 80 years of age and found that the incidence in this oldest age strata was consistently more than double that of patients aged 65‐79 years. These findings suggest that the impact of non‐influenza respiratory viruses is likely underappreciated in the absence of systematic testing of older adults hospitalized with acute respiratory signs and symptoms. Furthermore, lack of diagnostic testing could lead to lack of appropriate infection prevention and control and potential nosocomial transmission.

We found that influenza viruses predictably exhibited a high seasonal incidence with different types each year. The burden of non‐influenza respiratory viruses was potentially higher than that of influenza viruses. Non‐influenza respiratory viruses collectively had a higher incidence than that of influenza viruses collectively, although it is likely that the influenza vaccine reduced hospitalizations and mortality, particularly when there was a good match between the vaccine and circulating influenza strains.^12^ We found that 2.8 times more patients were hospitalized with non‐influenza viruses (excluding RV/EV) than with influenza viruses. Overall, the proportion of patients admitted to an ICU was significantly higher for non‐influenza than for influenza viruses. Mortality was also significantly higher for non‐influenza respiratory viruses. Notably, our analysis of the collective burden non‐influenza viruses could be perceived as an underestimate as we excluded RV/EV, which was the most commonly detected virus, as similarly described by others.^13^ We chose to exclude RV/EV from the analysis of the collective burden because RV/EV were the most commonly co‐detected viruses and the mPCR assay used cannot distinguish rhinovirus from enterovirus nor unique subtypes. Furthermore, while RV/EV are associated with hospitalizations for ARIs^14^ and exacerbations of underlying cardiac and pulmonary comorbidities,^13^, ^14^, ^15^ prolonged viral shedding is also well described,^16^ which could decrease the causal relationship of detecting these viruses with burden.

Comparison of our findings with other reports is challenging due to different methodologies including case finding and study population. For example, past studies exclusively examined older adults ≥ 65 years old and high‐risk adults with congestive heart failure and chronic obstructive pulmonary disorder.^6^ Furthermore, much of the morbidity and mortality associated with non‐influenza respiratory viruses has been described in adult long‐term care facilities experiencing outbreaks associated with high attack rates and high death rates.^17^, ^18^ Nonetheless, our incidence estimates of influenza hospitalizations were comparable to the Centers for Disease Control and Prevention (CDC) national estimates for both years.^19^, ^20^ The current study's rate of ICU admissions from RSV (16.1%) was similar to that described in previous prospective studies of RSV by Widmer and colleagues (10%)^3^ and Falsey and colleagues (15%).^6^ The current study's ICU admission rate of 11.5% associated with influenza was also similar to that previously described (6%,^3^ 12%^6^) as was the 16.5% rate associated with HMPV (13%^3^).

Some of our observations for the human coronaviruses merit further study as, to our knowledge, these findings have not been previously reported. We found a high burden associated with CoV 229E and CoV HKU1 as these (in addition to AV) had the highest rates of ICU admission and highest mortality rate. Among all the viruses studied, the combined CoV types had the highest incidence estimates for patients aged 18‐49 and 50‐64 years for both years. We found similar seasonality among the human coronavirus types as has been reported by the Centers for Disease Control and Prevention (CDC) and National Respiratory and Enteric Virus Surveillance System (NREVSS).^19^, ^20^, ^21^ However, the most common CoV type found by laboratories participating in NREVSS in 2018‐2019 was CoVOC43, while we found CoV229E and CoVOC43 to be the most common types that season. While surveillance reports from the NREVSS provide trends for and seasonality of respiratory viruses, these reports lack patient‐level characteristics to inform age‐related trends or patient outcomes. It remains to be seen whether seasonal variations in the human coronavirus will impact the epidemiology of the COVID‐19 pandemic or be a source of false‐positive serologic results for SARS‐CoV‐2 or even whether SARS‐CoV‐2 could eventually become a seasonal virus.

This study has limitations. It was performed at a university‐affiliated tertiary care referral center in New York City, which limits generalizability. We did not consider the impact of influenza vaccinations on hospitalization rate. Viral detections may not have represented acute infection as mPCR does not distinguish between viable and non‐viable virus. Case detection may have been decreased if not all patients were tested for respiratory viruses, particularly outside influenza season. However, the study period aligns with a prospective surveillance study of RSV in hospitalized adults conducted in the same facilities in which we found that nearly all hospitalized patients meeting ARI criteria during the respiratory viral season, October to April, were tested by treating clinicians.^22^ Finally, while the case definition required viral detection within 3 days of admission, we did not measure the burden of illness in cases that were acquired during hospitalization or in patients transferred to our hospital who had been tested at outside facilities.

In conclusion, the burden of non‐influenza respiratory viruses is substantial, particularly for older adults who lack durable immunity for respiratory viruses and frequently have comorbid conditions that increase their risk of severe disease. Thus, prevention and treatment strategies for non‐influenza respiratory viruses, including effective vaccines, are needed. Future research should further assess the clinical impact of specific human coronaviruses and the potential impact of specific coronavirus types on the epidemiology and impact of SARS‐CoV‐2.

## CONFLICT OF INTEREST

M. P. and L. F. Finelli are employees of Merck Sharp & Dohme Corp., a subsidiary of Merck & Co., Inc., Kenilworth, NJ, USA, and LS has received research funding from Merck & Co., Inc and served on an Advisory Board for Merck & Co., Inc

## AUTHOR CONTRIBUTIONS

**William D. Sieling:** Conceptualization (equal); data curation (lead); formal analysis (lead); investigation (lead); methodology (equal); software (lead); writing–original draft (lead); writing–review and editing (equal). **Connor R. Goldman:** Conceptualization (supporting); data curation (supporting); formal analysis (supporting); investigation (supporting); methodology (supporting); project administration (supporting); validation (supporting); writing–original draft (supporting); writing–review and editing (supporting). **Matthew Oberhardt:** Conceptualization (supporting); data curation (equal); formal analysis (equal); writing–review and editing (supporting). **Matthew Phillips:** Conceptualization (supporting); methodology (equal); project administration (equal); writing–review and editing (equal). **Lynn Finelli:** Conceptualization (equal); data curation (equal); formal analysis (equal); funding acquisition (equal); investigation (equal); methodology (equal); project administration (equal); writing–review and editing (equal). **Lisa Saiman:** Conceptualization (lead); data curation (lead); formal analysis (lead); funding acquisition (lead); investigation (lead); methodology (lead); writing–review and editing (lead).

### Peer Review

The peer review history for this article is available at https://publons.com/publon/10.1111/irv.12842.

## Data Availability

The data that support the findings of this study are available from the corresponding author upon reasonable request.
